# “Hallucinations” Following Acute Cannabis Dosing: A Case Report and Comparison to Other Hallucinogenic Drugs

**DOI:** 10.1089/can.2017.0052

**Published:** 2018-03-01

**Authors:** Frederick S. Barrett, Nicolas J. Schlienz, Natalie Lembeck, Muhammad Waqas, Ryan Vandrey

**Affiliations:** Behavioral Pharmacology Research Unit, Department of Psychiatry and Behavioral Sciences, Johns Hopkins University School of Medicine, Baltimore, Maryland.

**Keywords:** adverse event, cannabis, comparative pharmacology, hallucination, vaporization

## Abstract

**Introduction:** Cannabis has been historically classified as a hallucinogen. However, subjective cannabis effects do not typically include hallucinogen-like effects. Empirical reports of hallucinogen-like effects produced by cannabis in controlled settings, particularly among healthy research volunteers, are rare and have mostly occurred after administration of purified Δ-9 tetrahydrocannabinol (THC) rather than whole plant cannabis.

**Methods:** The case of a healthy 30-year-old male who experienced auditory and visual hallucinations in a controlled laboratory study after inhaling vaporized cannabis that contained 25 mg THC (case dose) is presented. Ratings on the Hallucinogen Rating Scale (HRS) following the case dose are compared with HRS ratings obtained from the participant after other doses of cannabis and with archival HRS data from laboratory studies involving acute doses of cannabis, psilocybin, dextromethorphan (DXM), and salvinorin A.

**Results:** Scores on the Volition subscale of the HRS were greater for the case dose than for the maximum dose administered in any other comparison study. Scores on the Intensity and Perception subscales were greater for the case dose than for the maximum dose of cannabis, psilocybin, or salvinorin A. Scores on the Somaesthesia subscale were greater for the case dose than for the maximum dose of DXM, salvinorin A, or cannabis. Scores on the Affect and Cognition subscales for the case dose were significantly lower than for the maximum doses of psilocybin and DXM.

**Conclusion:** Acute cannabis exposure in a healthy adult male resulted in self-reported hallucinations that rated high in magnitude on several subscales of the HRS. However, the hallucinatory experience in this case was qualitatively different than that typically experienced by participants receiving classic and atypical hallucinogens, suggesting that the hallucinatory effects of cannabis may have a unique pharmacological mechanism of action. This type of adverse event needs to be considered in the clinical use of cannabis.

## Introduction

Cannabis, containing the psychoactive constituent Δ-9 tetrahydrocannabinol (THC), was historically classified as a hallucinogen, possibly due to the observation of powerful psychoactive effects. Early case reports that documented the subjective effects of cannabis^1,2^ included experiences of anxiety, physiological distress, spiritual or mystical effects, and alterations to perception, awareness, and insight. These case reports provided support for characterizing cannabis as a hallucinogen.^2^ Currently, it is uncommon for cannabis to be categorized as a hallucinogen, but, as policy changes regarding the medicinal and nonmedicinal (i.e., “recreational”) use of cannabis are rapidly being implemented, revisiting this potential effect of cannabis is warranted.

Hallucinations may be elicited by a variety of psychoactive drugs, with variable pharmacology. That said, hallucinations are consistently observed, and, arguably, the defining feature of a subset of drugs. The primary subjective effects of classic hallucinogens (e.g., psilocybin, lysergic acid diethylamide, and dimethyltryptamine [DMT]) are altered perception and cognition,^3^ mystical or spiritual experiences,^4,5^ and occasional anxiety and physiological distress,^6^ and the molecular mechanism of action of hallucinogens is understood to be 5HT_2A_ receptor agonism.^7,8^ A set of pharmacologically diverse atypical hallucinogens, including κ-opioid agonists (e.g., salvinorin A)^9^ and N-methyl-D-aspartate (NMDA) antagonists (including ketamine and dextromethorphan [DXM]),^10^ are also known to consistently produce effects similar to, but distinguishable from, those of classic hallucinogens. THC is a partial agonist of cannabinoid receptor type-1 (CB1) and type-2 (CB2) receptors. Neither THC, nor minor cannabinoids, nor the terpenoids present in cannabis plant material have known direct effects at the 5HT_2A_, κ-opioid, or NMDA receptor.^11^

Most case reports describing hallucinations following acute cannabis exposure involve individuals with current psychosis or a family history of psychosis, populations that are known to have an atypical response to cannabis.^12^ A growing literature continues to explore the relationship between cannabis use and development of psychosis among individuals with an underlying vulnerability for psychosis.^13–16^ While perceptual alterations in healthy individuals during the acute effects of THC have been described,^17,18^ empirical reports describing hallucinogen-like effects of cannabis and cannabis constituents in controlled settings and in healthy participants without a family history of psychosis are quite rare.

Peer-reviewed reports that do detail hallucinogen-like experiences in healthy adults predominantly come from research studies involving the administration of purified THC^16^ or case reports of individuals who experience adverse reactions following use of synthetic cannabinoids (often full CB1 agonists with greater potency than THC),^19^ rather than whole plant cannabis. This distinction is worth noting as it points to exogenous CB1 receptor agonism as a potential mechanism for inducing hallucinations, and also because it has been postulated that phytocannabinoids such as cannabidiol (CBD)^20^ or terpenoids^11^ that are present in the cannabis plant may mitigate some of the deleterious effects of THC.

The following report presents the case of an atypical response to cannabis that included self-reported “hallucinogen-like” effects after inhalation of vaporized cannabis containing THC, but nominal levels of CBD in a controlled laboratory study. To qualitatively investigate the phenomenology of this hallucinogen-like experience, we conducted cross-sectional comparisons of data from this case with archival data obtained from controlled behavioral pharmacology studies of cannabis, the classic hallucinogen psilocybin, and the atypical hallucinogens DXM and salvinorin A.

## Methods

### Approach

In the context of a double-blind, placebo-controlled laboratory study investigating the effects of smoked and vaporized cannabis, we present the details of an adverse reaction to inhalation of vaporized cannabis containing 25 mg THC (the “case dose”) in a healthy male research participant (H.C.). Ratings of subjective drug effects provided by H.C. during the case dose are compared with subjective data obtained from other participants in the same study, as well as subjective effects reported by participants in separate self-administration studies that evaluated acute dose effects of oral cannabis, psilocybin,^5^ DXM,^10^ and salvinorin A.^9,21^

### Study methods

All research studies were conducted at the Johns Hopkins Behavioral Pharmacology Research Unit (BPRU). Across investigations, research participants were enrolled if they were medically healthy adults who screened negative for current Axis I psychiatric disorders, denied a personal or family history of psychosis (i.e., first or second-degree relative), did not meet formal diagnostic criteria for substance use disorders (other than caffeine or tobacco), and were not taking medications that could interact with study drugs. Urine drug tests verified abstinence from drugs of abuse before all experimental sessions.

#### Cannabis studies

Subjective effects data from 31 healthy adult participants were collected across two cannabis self-administration studies; one experiment (*N*=17) evaluated acute doses of orally ingested cannabis in brownies that contained 0, 10, 25, and 50 mg THC, and a second study (*N*=14), in which H.C. was a participant, examined acute doses of smoked, and vaporized cannabis containing 0, 10, or 25 mg THC. Cannabis plant matter was sourced from and certified by the Drug Supply Program of the National Institute on Drug Abuse (NIDA) to contain 13.4% THC and 0.03% CBD. Dried cannabis was weighed and dispensed in a quantity that was calculated to contain the target dose of THC (e.g., 186.6 mg of cannabis would be vaporized to deliver 25 mg THC). Participants endorsed a history of cannabis use and denied use of cannabis for at least 30 days before study enrollment. Participants were not dependent on or seeking treatment for cannabis or other psychoactive drugs. A minimum of 1 week separated each dose condition to allow for a full washout of doses, and washout was biochemically verified with quantitative urine and blood toxicology tests.

#### Psilocybin study

Eighteen healthy adult participants, 17 who were hallucinogen-naive, completed five experimental sessions involving administration of 0, 5, 10, 20, and 30 mg/70 kg psilocybin doses in a controlled and supportive laboratory setting.^5^ Drug administration sessions were separated by ∼1 month. Data collected during the 5 mg/70 kg dose is not included in this report.

#### DXM study

Twelve healthy adults with histories of hallucinogen use were administered up to eight doses of DXM (100–800 mg/70 kg), two doses of the sedative–hypnotic drug triazolam (0.25 and 0.5 mg/70 kg), and placebo under blinded conditions using an ascending dose run-up design.^10^ A minimum of 48 h separated each drug administration session. All participants received at least the first four doses of DXM (100, 200, 300, and 400 mg/70 kg), but the study was halted before the 800 mg/70 kg dose for 10 participants due to adverse effects at lower doses. For the current comparison, we present data from placebo, 200 mg, the second highest (penultimate) dose, and the maximum dose administered to each participant.

#### Salvinorin A study

Eight healthy adults with previous lifetime use of a classic hallucinogen and at least one instance of *salvia divinorum* use in the past 5 years inhaled up to 16 ascending doses of vaporized salvinorin A (0.375–21 μg/kg).^9^ A minimum of 24 h separated each drug administration session. Data from placebo, 9, 15, and 19.5 μg/kg doses were used for comparison in this study, as these doses of salvinorin A roughly corresponded to low, moderate, and high doses of psilocybin^5^ on ratings of drug effect intensity.

### Hallucinogen effects assessment

Participants across studies completed the Hallucinogen Rating Scale (HRS).^22^ The HRS has been widely used to investigate the effects of a range of classic hallucinogens, including DMT,^23–26^ psilocybin,^4,5^ 2C-B,^27^ and 3,4-methylenedioxy-N-ethylamphetamine.^28^ The HRS contains 59 items that are rated using a 5-point scale (0—not at all, 1—slightly, 2—moderately, 3—very much, 4—extremely) and scored with subscales indicating the degree of change that occurs during an acute drug experience from a more typical everyday experience across six dimensions (with example items for each dimension): *Intensity* (“high,” “a rush”), *Somaesthesia* (“change in body temperature,” “electric/tingling feeling”), *Affect* (“panic,” “euphoria”), *Perception* (“change in distinctiveness of sounds,” “change in brightness of objects in room”), *Cognition* (“change in rate of thinking,” “change in quality of thinking”), and *Volition* (“in control,” “able to move around if asked to”). Subscale scores were calculated as the average rating on all items that load onto each subscale.

### Analyses

Average scores from the HRS were computed for each drug condition in each study, and plotted along with the HRS scores from responses for each dose and route of administration reported by H.C. A one-sample Student's *t*-test was used to compare HRS scores from each drug condition in each study to the HRS scores for H.C. during the experimental session (vaporized cannabis containing 25 mg THC) in which he self-reported experiencing hallucinations.

## Results

### Case description

H.C. presented as a medically and psychiatrically healthy 30-year-old Caucasian male. He denied a history of significant health and psychiatric conditions and denied a family history of psychosis. He endorsed prior cannabis use, denied a history of any significant adverse effects associated with prior use, and he disclosed that 6 years had passed since he last used cannabis. He reported weekly use of alcohol and caffeine and denied use of nicotine/tobacco products and illicit drugs. He endorsed use of over-the-counter medication as needed for seasonal allergies. During the first three study sessions, he smoked cannabis that contained 0, 10, or 25 mg THC through hand-held pipe. Dose-related subjective drug effects, cardiovascular effects, and impairment on cognitive performance assessments were observed as expected. On the fourth experimental session (as with other experimental sessions), baseline assessments were within normal limits and urine drug screening (for common drugs of abuse) and breath alcohol tests were negative. After consuming a standard low-fat breakfast, the participant self-administered vaporized cannabis that contained 25 mg THC within 10 min (per protocol). Acute drug effects escalated in magnitude for the first 20 min following inhalation. He had difficulty responding to staff inquiries, was unable to complete self-report questionnaires, had difficulty keeping his head up, and appeared to periodically fall asleep or lose consciousness despite encouragement by research staff to stay awake and continue. He was unable to maintain a balanced, steady gait when he walked.

H.C. displayed behavior consistent with heavy sedation. The volunteer had difficulty maintaining consciousness and, at times, would not respond to verbal inquiries by study staff. He was under direct supervision of medical staff and neither his vital signs nor his behavior required medical intervention. He was able to complete a self-reported drug effect questionnaire, but had extreme difficulty completing cognitive performance assessments in the first 90 min following drug exposure. When he did speak, he reported feeling faint, dizzy, nauseated and that he was experiencing tingling sensations in his arms and legs and pain at the base of his neck.

Quantitative analysis (LC/MS/MS) of whole blood collected 10 min after the completion of cannabis administration (peak level measured in this study) showed 16 ng/mL THC, 3 ng/mL 11-OH-THC, and 17 ng/mL THC-COOH for H.C. These are consistent with mean values (14 ng/mL THC, 2 ng/mL 11-OH-THC, and 7 ng/mL THC-COOH) observed for all participants in this study at that time point and dose of vaporized cannabis.^29^ Analysis of whole blood collected 10 min after the completion of cannabis administration in the smoked condition for the same dose level for H.C. (25 mg THC) showed 1 ng/mL THC, 1 ng/mL 11-OH-THC, and 4 ng/mL THC-COOH, and these values are consistent with mean values observed for all participants in this study at that time point and dose of smoked cannabis.^29^

Three hours after drug administration, his symptoms began to decrease in severity. He indicated that he had experienced a dissociative state and altered perceptions of auditory and visual stimuli at the time of peak drug effect. He reported a hypersensitivity to voices at that time, which he described as if he was more aware of conversations around him, but was unable to hear or understand distinct words. He described visual distortions in the form of the environment and floor sinking away and the appearance of patterns moving on the carpet and chairs in the room. Additionally, he reported an “out-of-body” experience characterized by the feeling of being removed from his body, existing above it in space, and feeling that his surroundings were sinking away from him, which was also accompanied by a feeling of paralysis. He reported having had a similar experience when administered ketamine before surgery for a broken leg. Four hours after drug administration, and after eating lunch, H.C.'s symptoms of nausea, faintness, dissociation, and auditory, visual, and perceptual alterations had almost completely subsided. Five hours after drug administration, he appeared more alert and was able to complete all study-related tasks.

At the end of the experimental session, H.C. was prompted to recount his experience. He reported feeling overwhelmed that it was an uncomfortable, scary, and unpleasant experience akin to what he would expect an overdose or anxiety attack may feel like, and he indicated he never wanted to have the experience again. He expressed the sense that he thought he would “never come out of this” and that he would always feel the adverse effects. Within 8 h of acute drug administration, measures of cognitive performance, subjective mood and drug effects assessments, and vital signs had returned to baseline levels. The study medical team determined that there was no significant health risk with continued study participation and H.C. completed two remaining experimental sessions (lower dose and placebo cannabis) without significant discomfort.

### Comparison of Hallucinogen Rating Scale scores

**FIG. 1.**
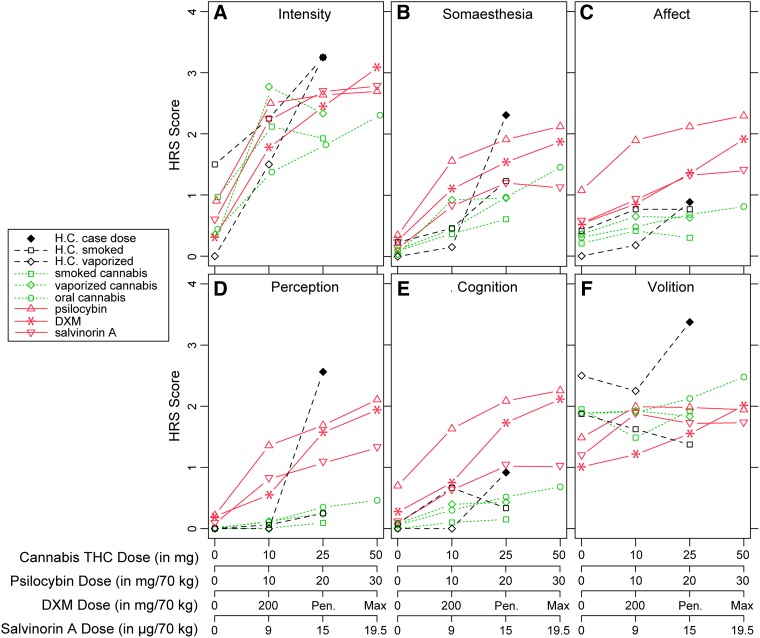
Comparison of HRS scores across studies. **(A)** HRS Intensity score, **(B)** HRS Somaesthesia score, **(C)** HRS affect score, **(D)** HRS perception score, **(E)** HRS cognition score, and **(F)** HRS volition score. Each **(A–F)** compares the scores for the indicated scale of the HRS (ordinate) at each of four dose conditions for each drug (abscissa) for each of the studies identified within the legend. Pen=average of ratings provided after the second-highest (penultimate) dose of DXM that a given participant received.^10^ Max=average of ratings provided after the highest dose of DXM that a given participant received.^10^ “Cannabis THC dose” refers to the amount of THC contained in dried cannabis that was administered. Smoked cannabis and vaporized cannabis studies are not yet published. HRS, Hallucinogen Rating Scale; THC, tetrahydrocannabinol.

The participant's scores on the HRS are plotted in Figure 1 along with mean participant HRS scores from studies of acute cannabis, psilocybin, DXM, and salvinorin A drug administration. Volition scores (Fig. 1F) were significantly greater for the participant's 25 mg THC dose of vaporized cannabis (referred to as “the case dose”) than for mean scores following the maximum dose of all other drug conditions and studies (all *p*s<0.0001). Intensity scores (Fig. 1A) from the case dose were not significantly different than mean Intensity scores from the maximum tolerated dose (400–800 mg) of DXM [*t*(11)=1.21, *p*=0.13], but were significantly greater than the mean Intensity scores for the maximally rated dose of psilocybin [30 mg/70 kg; *t*(17)=4.55, *p*<0.0005], salvinorin A [19.5 μg/kg; *t*(7)=−2.57, *p*<0.05], smoked cannabis [25 mg THC; *t*(9)=−5.69, *p*<0.0005], vaporized cannabis [25 mg THC; *t*(11)=−3.93, *p*<0.005], and oral cannabis [50 mg THC; *t*(15)=−3.71, *p*<0.005]. The score on the Perception subscale (Fig. 1D) of the HRS for the case dose was qualitatively higher than mean scores following the maximum tolerated dose (400–800 mg/70 kg) of DXM [*t*(11)=1.78, *p*=0.051], and was significantly greater than those for the maximum dose of psilocybin [30 mg/70 kg; *t*(17)=−2.24, *p*<0.05], salvinorin A [19.5 μg/kg; *t*(7)=−5.99, *p*<0.0005], and all cannabis conditions (*p*<0.0001). HRS scores for the Somaesthesia subscale (Fig. 1B) were not significantly different between the case dose and the maximum dose of psilocybin [30 mg/70 kg; *t*(17)=−1.24, *p*=0.23], but Somaesthesia was significantly greater for the case dose than for the maximum tolerated dose (400–800 mg/70 kg) of DXM [*t*(11)=1.90, *p*<0.05] as well as the maximum dose of salvinorin A [19.5 μg/kg; *t*(7)=−7.2, *p*<0.0001], and all cannabis doses (*p*<0.001).

The Affect score (Fig. 1C) for the case dose was significantly lower than the Affect score for the 30 mg/70 kg psilocybin dose [*t*(17)=8.31, *p*<0.00001], the 10 mg/70 kg psilocybin dose [*t*(17)=6.22, *p*<0.00001], and the maximum tolerated dose of DXM [400–800 mg/70 kg; *t*(11)=4.11, *p*<0.001], but not the 200 mg/70 kg dose of DXM, the maximum dose of salvinorin A, or any of the cannabis conditions (all *p*s>0.13). The Cognition score (Fig. 1E) for the case dose was also significantly lower than the Cognition score for the 30 mg/70 kg psilocybin dose [*t*(17)=6.12, *p*<0.00001], the 10 mg/70 kg psilocybin dose [*t*(17)=4.05, *p*<0.001], and the maximum tolerated dose of DXM [400–800 mg/70 kg; *t*(11)=3.62, *p*<0.005], but not the 200 mg/70 kg dose of DXM, the maximum dose of salvinorin A, or any of the cannabis conditions (all *p*s>0.17).

## Discussion

The legalization of cannabis for medicinal and nonmedicinal use is rapidly expanding. The development of novel routes of administration and technologies for delivering cannabis raises concerns about the adequacy of available data to inform dosing recommendations and complete disclosure of potential adverse consequences. In a controlled research study, a 30-year-old healthy male research participant (H.C.) experienced an adverse reaction to an acute dose of vaporized cannabis containing 25 mg of THC self-administered over the course of 10 min. H.C.'s response appeared somewhat remarkable in that he reported distortions in visual and auditory perception, cognition, and volition. These effects are not typical in controlled laboratory studies of healthy adults who do not report prior adverse reactions to cannabis or family history of psychosis. He reported large changes on the Intensity, Somaesthesia, Perception, and Volition subscales of the HRS, domains associated with classical hallucinogen drug effects, but relatively low ratings on the Affect and Cognition subscales compared with HRS ratings obtained in controlled laboratory studies of other hallucinogenic drugs. The case dose was greater in Intensity and Perception subscales of the HRS compared with mean scores obtained following administration of high doses of psilocybin and salvinorin A, but was comparable to Perception scores following the maximum tolerated dose of DXM.^10^ This is consistent with the participant's report of a ketamine-like experience, given that DXM is a dissociative hallucinogen/anesthetic with a similar mechanism of action to ketamine (NMDA antagonism). The participant's scores on the Somaesthesia subscale were similar to those observed after administration of a high dose of oral psilocybin (30 mg/70 kg),^5^ both of which were greater than scores observed following DXM and salvinorin A administration. Scores on the Affect and Cognition subscales of the HRS for the case dose were low, consistent with scores provided by other participants in acute cannabis dosing studies, and were not consistent with the most intense reported experiences with psilocybin, DXM, or salvinorin A.

Given that changes in affect and cognition are core features of experience with classic hallucinogens,^3^ it is difficult to attribute the reported case as an experience similar to that of a true classic hallucinogen. Curiously, Intensity scores for the case dose were equal to the Intensity score for H.C. after self-administration of smoked cannabis containing the 25 mg THC dose. H.C. did not report any hallucinations during the 25 mg THC smoked cannabis session. Also, scores on the Volition subscale for all vaporized drug conditions, including placebo, were greater than for sessions involving smoked cannabis for H.C., and were higher than those observed in archival study comparisons.

A number of case reports have been recently published that indicate psychotic or hallucinogen-like effects after ingestion of synthetic cannabinoids in both adults^19,30,31^ and adolescents.^32,33^ In many cases, these individuals, like H.C., were otherwise healthy, had negative toxicology screens for other substances of abuse, and were typically free of personal or family history of psychosis.^31^ Unlike H.C., cases reported after consumption of synthetic cannabinoids included seizures, agitation or violent behavior, and frank psychosis,^19^ or psychiatric syndromes persisting for days or longer after consumption of synthetic cannabinoids.^31^ One study reported development of hallucinogen persistent perceptual disorder (HPPD) after synthetic cannabinoid use in otherwise healthy adults who had no prior history of natural or synthetic hallucinogen use^30^; however, H.C. encountered no persisting effects, either HPPD or other, which were related to the case dose in this report. While THC is a partial agonist of the CB1 receptor, synthetic cannabinoids tend to be full CB1 agonists with high potency and high affinity for the CB1 receptor. These factors may contribute to a greater likelihood (compared with cannabis) of negative and hallucinogen-like effects after consumption of synthetic cannabinoids, and may suggest that CB1 agonism may underlie hallucinogen-like effects of cannabinoids.^34^ While H.C.'s experience was atypical for a response to cannabis or THC, it does not seem that his experience is consistent with published case reports of “hallucinogenic” effects following consumption of synthetic cannabinoids.

Although the subjective effects that H.C. reported exhibited some similarities to the effects of classic and atypical hallucinogens (Fig. 1), the overall profile of subjective effects as characterized by the HRS indicates that his experience was not wholly consistent with what would be expected for a classic hallucinogen, an NMDA antagonist dissociative hallucinogen (DXM, similar in effects to ketamine), or κ-opioid agonist (salvinorin A). Thus, it appears that the hallucinatory effects of cannabis, taken as a whole, may be qualitatively different than those of other hallucinogens, which suggests that the hallucinatory effects of cannabis may have a unique pharmacological mechanism of action.

### Potential mechanisms of hallucinatory effects of cannabis

Although cannabis constituents do not have high affinity for direct pharmacological effects at 5HT_2A_, NMDA, or κ-opioid receptors, they may interact with the serotonin and glutamatergic systems. Multiple preclinical studies have demonstrated an impact of exogenous cannabinoid administration on 5-HT receptor expression and function3. CBD has been shown to alter psychological response to ketamine,^35^ and subchronic administration of PCP (an NMDA antagonist) has been shown to change the effects of exogenous cannabinoids on prefrontal brain function.^36^ However, existing studies suggest that the administration of exogenous cannabinoids does not modulate the κ-opioid receptor system.^37–39^ While speculative, it is possible that hallucinatory effects of cannabinoids may be due to effects of exogenous cannabinoid on 5-HT or NMDA receptors; however, studies explicitly evaluating cannabinoid effects in brain areas associated with hallucinations are lacking.

### Potential factors contributing to individual differences in cannabinoid response

Blood cannabinoid levels measured after the case dose were comparable to means observed in other study participants for vaporized administration of cannabis containing the same dose of THC (25 mg). Similarly, blood cannabinoid levels measured for H.C. after smoked administration of cannabis containing a dose of THC equal to the case dose were far lower than those measured after the case dose, consistent with means observed in other study participants. This is consistent with suggests that H.C. was not exposed to a greater amount of THC than other study participants. Given H.C.'s history of a strong reaction to a psychoactive and potentially hallucinogenic drug (ketamine) in a clinical setting, this suggests that H.C. may possess some unique sensitivity to psychoactive drugs.

Additional research is needed to help understand the neurobiological underpinnings of hallucinations that are sometimes occasioned following high-dose cannabis administration. For example, there may be genetic differences between individuals who experience and those who do not experience rare and atypical effects of cannabinoids that account for these effects. Although we did not sample and cannot address the genetic profile of H.C., it is possible that genes implicated in cannabis-induced psychosis (such as the DRD2, BDNF, AKT1, and COMT genes)^40^ are also involved in the type and degree of experience that was encountered in this case. In addition, individual differences in sensitivity to CB1 agonists (potentially mediated by the CNR1 gene) may predict individual differences in response to exogenous cannabinoids. As mentioned previously, there are hypotheses that cannabis constituents other than THC (e.g., CBD, other phytocannabinoids, or terpenoids) may mitigate some of the adverse effects of THC. The cannabis used in the present study contained a high concentration of THC (13%) and low concentrations of CBD (<1%) and CBN (<1%). While an extreme ratio of THC to these minor phytocannabinoids may increase the likelihood of adverse events such as the hallucinations observed in this report, there is insufficient empirical data on the interaction between THC and other constituents of the plant to confidently draw that conclusion at this point. It is also important to note that most research evaluating the cannabinoid profile of commercial cannabis and related products indicate that very high THC and very low CBD products are predominant in the current retail market.^41–43^

### Limitations

The current report describes perceptual alterations and dissociative symptoms of a type that have been sparsely described previously as resulting from acute cannabis exposure. There is a substantial literature pointing to cannabinoid-induced psychosis in those with a personal or family history of psychosis. This particular susceptibility to psychosis was nominally ruled out in the case of H.C. in the medical and psychiatric history collected during screening, but the participant may have misreported or there may be a latent family history of which he was unaware. The current article is also limited in that cross-study comparisons with respect to the qualitative and quantitative characteristics of hallucinations across drug types was conducted with archival data across subjects rather than prospective evaluation within the same individuals. Additional research on the comparative effects of these drugs, especially those that incorporate genetic and neuroimaging components, is needed to extend the present observations.

## Conclusion

In this article, we highlighted a rare but clinically significant response to acute cannabis dosing. The participant was functionally incapacitated for about 90 min and experienced strongly aversive and disorienting effects. It is unclear how frequently this type of reaction occurs in healthy adults without a family history of psychosis. H.C. did not have any health screening information that would have predicted this effect, indicating that this type of reaction should be considered in decision making regarding cannabis use. This case also demonstrates the importance of considering dose and route of administration in decision making regarding cannabis use as this individual did not exhibit similar effects at lower vaporized doses or smoking the same dose of cannabis.

## Abbreviations Used

BPRU: Behavioral Pharmacology Research Unit
CB1: cannabinoid receptor type-1
CBD: cannabidiol
DMT: dimethyltryptamine
DXM: dextromethorphan
HPPD: hallucinogen persistent perceptual disorder
HRS: Hallucinogen Rating Scale
NIDA: National Institute on Drug Abuse
NMDA: N-methyl-D-aspartate
THC: tetrahydrocannabinol

## References

[1] Perna D Psychotogenic effect of marihuana. JAMA. 1969;209:1085–1086 5819660

[2] Keeler MH, Ewing JA, Rouse BA Hallucinogenic effects of marijuana as currently used. Am J Psychiatry. 1971;128:213–216 5112948 10.1176/ajp.128.2.213

[3] Preller KH, Vollenweider FX Phenomenology, structure, and dynamic of psychedelic states. Curr Top Behav Neurosci. 2016 [Epub ahead of print]; DOI: 10.1007/7854_2016_459 28025814

[4] Griffiths RR, Richards WA, McCann U, et al. Psilocybin can occasion mystical-type experiences having substantial and sustained personal meaning and spiritual significance. Psychopharmacology (Berl). 2006;187:268–283 16826400 10.1007/s00213-006-0457-5

[5] Griffiths RR, Johnson MW, Richards WA, et al. Psilocybin occasioned mystical-type experiences: immediate and persisting dose-related effects. Psychopharmacology (Berl). 2011;218:649–665 21674151 10.1007/s00213-011-2358-5 PMC3308357

[6] Barrett FS, Bradstreet MP, Leoutsakos JS, et al. The challenging experience questionnaire: characterization of challenging experiences with psilocybin mushrooms. J Psychopharmacol. 2016;30:1279–1295 27856683 10.1177/0269881116678781 PMC5549781

[7] Nichols DE Psychedelics. Pharmacol Rev. 2016;68:264–355 26841800 10.1124/pr.115.011478 PMC4813425

[8] Halberstadt AL Recent advances in the neuropsychopharmacology of serotonergic hallucinogens. Behav Brain Res. 2015;277:99–120 25036425 10.1016/j.bbr.2014.07.016 PMC4642895

[9] MacLean KA, Johnson MW, Reissig CJ, et al. Dose-related effects of salvinorin A in humans: dissociative, hallucinogenic, and memory effects. Psychopharmacology (Berl). 2013;226:381–392 23135605 10.1007/s00213-012-2912-9 PMC3581702

[10] Reissig CJ, Carter LP, Johnson MW, et al. High doses of dextromethorphan, an NMDA antagonist, produce effects similar to classic hallucinogens. Psychopharmacology (Berl). 2012;223:1–15 22526529 10.1007/s00213-012-2680-6 PMC3652430

[11] Russo EB Taming THC: potential cannabis synergy and phytocannabinoid-terpenoid entourage effects. Br J Pharmacol. 2011;163:1344–1364 21749363 10.1111/j.1476-5381.2011.01238.x PMC3165946

[12] D'Souza DC, Abi-Saab WM, Madonick S, et al. Delta-9-tetrahydrocannabinol effects in schizophrenia: implications for cognition, psychosis, and addiction. Biol Psychiatry. 2005;57:594–608 15780846 10.1016/j.biopsych.2004.12.006

[13] Curran HV, Freeman TP, Mokrysz C, et al. Keep off the grass? Cannabis, cognition and addiction. Nat Rev Neurosci. 2016;17:293–306 27052382 10.1038/nrn.2016.28

[14] Gage SH, Hickman M, Zammit S Association between cannabis and psychosis: epidemiologic evidence. Biol Psychiatry. 2016;79:549–556 26386480 10.1016/j.biopsych.2015.08.001

[15] Hanna RC, Perez JM, Ghose S Cannabis and development of dual diagnoses: a literature review. Am J Drug Alcohol Abuse. 2017;43:442–455 27612527 10.1080/00952990.2016.1213273 PMC5344774

[16] Sherif M, Radhakrishnan R, D'Souza DC, et al. Human laboratory studies on cannabinoids and psychosis. Biol Psychiatry. 2016;79:526–538 26970363 10.1016/j.biopsych.2016.01.011

[17] Tart CT Marijuana intoxication common experiences. Nature. 1970;226:701–704 5443246 10.1038/226701a0

[18] Winton-Brown TT, Allen P, Bhattacharyya S, et al. Modulation of auditory and visual processing by delta-9-tetrahydrocannabinol and cannabidiol: an FMRI study. Neuropsychopharmacology. 2011;36:1340–1348 21412224 10.1038/npp.2011.17 PMC3096803

[19] Harris CR, Brown A Synthetic cannabinoid intoxication: a case series and review. J Emerg Med. 2013;44:360–366 22989695 10.1016/j.jemermed.2012.07.061

[20] Niesink RJ, van Laar MW Does cannabidiol protect against adverse psychological effects of THC? Front Psychiatry. 2013;4:13 0 24137134 10.3389/fpsyt.2013.00130 PMC3797438

[21] Johnson MW, MacLean KA, Reissig CJ, et al. Human psychopharmacology and dose-effects of salvinorin A, a kappa opioid agonist hallucinogen present in the plant *salvia divinorum*. Drug Alcohol Depend. 2011;115:150–155 21131142 10.1016/j.drugalcdep.2010.11.005 PMC3089685

[22] Strassman RJ, Qualls CR, Uhlenhuth EH, et al. Dose-response study of N,N-dimethyltryptamine in humans. II. subjective effects and preliminary results of a new rating scale. Arch Gen Psychiatry. 1994;51:98–108 8297217 10.1001/archpsyc.1994.03950020022002

[23] Riba J, Rodriguez-Fornells A, Urbano G, et al. Subjective effects and tolerability of the South American psychoactive beverage ayahuasca in healthy volunteers. Psychopharmacology (Berl). 2001;154:85–95 11292011 10.1007/s002130000606

[24] Riba J, Anderer P, Jane F, et al. Effects of the South American psychoactive beverage ayahuasca on regional brain electrical activity in humans: a functional neuroimaging study using low-resolution electromagnetic tomography. Neuropsychobiology. 2004;50:89–101 15179026 10.1159/000077946

[25] Bouso JC, Gonzalez D, Fondevila S, et al. Personality, psychopathology, life attitudes and neuropsychological performance among ritual users of ayahuasca: a longitudinal study. PLoS One. 2012;7:e4242 1 10.1371/journal.pone.0042421 PMC3414465 22905130

[26] Bogenschutz MP, Forcehimes AA, Pommy JA, et al. Psilocybin-assisted treatment for alcohol dependence: a proof-of-concept study. J Psychopharmacol. 2015;29:289–299 25586396 10.1177/0269881114565144

[27] Caudevilla-Galligo F, Riba J, Ventura M, et al. 4-bromo-2,5-dimethoxyphenethylamine (2C-B): presence in the recreational drug market in spain, pattern of use and subjective effects. J Psychopharmacol. 2012;26:1026–1035 22234927 10.1177/0269881111431752

[28] Gouzoulis-Mayfrank E, Thelen B, Habermeyer E, et al. Psychopathological, neuroendocrine and autonomic effects of 3,4-methylenedioxyethylamphetamine (MDE), psilocybin and d-methamphetamine in healthy volunteers. Results of an experimental double-blind placebo-controlled study. Psychopharmacology (Berl). 1999;142:41–50 10102781 10.1007/s002130050860

[29] Schlienz NJ, Cone EJ, Herrmann ES, et al. Comparative pharmacodynamic investigation of oral, smoked, and vaporized cannabis. 2017 Available at http://www.icrs.co/SYMPOSIUM.2017/ICRS2017.FINAL.PROGRAMME.pdf (accessed 326, 2018)

[30] G Lerner A, Goodman C, Bor O, et al. Synthetic cannabis substances (SPS) use and hallucinogen persisting perception disorder (HPPD): two case reports. Isr J Psychiatry Relat Sci. 2014;51:277–280 25841224

[31] Hurst D, Loeffler G, McLay R Psychosis associated with synthetic cannabinoid agonists: a case series. Am J Psychiatry. 2011;168:111 9 21969050 10.1176/appi.ajp.2011.11010176

[32] Castellanos D, Singh S, Thornton G, et al. Synthetic cannabinoid use: a case series of adolescents. J Adolesc Health. 2011;49:347–349 21939863 10.1016/j.jadohealth.2011.08.002

[33] Besli GE, Ikiz MA, Yildirim S, et al. Synthetic cannabinoid abuse in adolescents: a case series. J Emerg Med. 2015;49:644–650 26293411 10.1016/j.jemermed.2015.06.053

[34] van Amsterdam J, Brunt T, van den Brink W The adverse health effects of synthetic cannabinoids with emphasis on psychosis-like effects. J Psychopharmacol (Oxford). 2015;29:254–263 25586398 10.1177/0269881114565142

[35] Hallak JEC, Dursun SM, Bosi DC, et al. The interplay of cannabinoid and NMDA glutamate receptor systems in humans: preliminary evidence of interactive effects of cannabidiol and ketamine in healthy human subjects. Prog Neuropsychopharmacol Biol Psychiatry. 2011;35:198–202 21062637 10.1016/j.pnpbp.2010.11.002

[36] Aguilar DD, Giuffrida A, Lodge DJ THC and endocannabinoids differentially regulate neuronal activity in the prefrontal cortex and hippocampus in the subchronic PCP model of schizophrenia. J Psychopharmacol (Oxford). 2016;30:169–181 26510449 10.1177/0269881115612239 PMC5252830

[37] Solinas M, Goldberg SR Involvement of mu-, delta- and kappa-opioid receptor subtypes in the discriminative-stimulus effects of delta-9-tetrahydrocannabinol (THC) in rats. Psychopharmacology (Berl). 2005;179:804–812 15619107 10.1007/s00213-004-2118-x

[38] Berrendero F, Mendizábal V, Murtra P, et al. Cannabinoid receptor and WIN 55 212-2-stimulated [35S]-GTPgammaS binding in the brain of mu-, delta- and kappa-opioid receptor knockout mice. Eur J Neurosci. 2003;18:2197–2202 14622180 10.1046/j.1460-9568.2003.02951.x

[39] Kathmann M, Flau K, Redmer A, et al. Cannabidiol is an allosteric modulator at mu- and delta-opioid receptors. Naunyn Schmiedebergs Arch Pharmacol. 2006;372:354–361 16489449 10.1007/s00210-006-0033-x

[40] Silveira MM, Arnold JC, Laviolette SR, et al. Seeing through the smoke: human and animal studies of cannabis use and endocannabinoid signalling in corticolimbic networks. Neurosci Biobehav Rev. 2017;76:380–395 27639448 10.1016/j.neubiorev.2016.09.007 PMC5350061

[41] Vandrey R, Raber JC, Raber ME, et al. Cannabinoid dose and label accuracy in edible medical cannabis products. JAMA. 2015;313:2491–2493 26103034 10.1001/jama.2015.6613

[42] Niesink RJ, Rigter S, Koeter MW, et al. Potency trends of Δ9-tetrahydrocannabinol, cannabidiol and cannabinol in cannabis in the netherlands: 2005–2015. Addiction. 2015;110:1941–1950 26234170 10.1111/add.13082

[43] Mammen G, de Freitas L, Rehm J, et al. Cannabinoid concentrations in canada's regulated medical cannabis industry. Addiction. 2017;112:730–732 28124470 10.1111/add.13738

